# Child mental health differences amongst ethnic groups in Britain: a systematic review

**DOI:** 10.1186/1471-2458-8-258

**Published:** 2008-07-25

**Authors:** Anna Goodman, Vikram Patel, David A Leon

**Affiliations:** 1 Department of Epidemiology and Population Health, London School of Hygiene and Tropical Medicine, London WC1E 7HT, UK

## Abstract

**Background:**

Inter-ethnic differences have been reported for many mental health outcomes in the UK, but no systematic review on child mental health has been published. The aim of this review is to compare the population-based prevalence of child mental disorders between ethnic groups in Britain, and relate these findings to ethnic differences in mental health service use.

**Methods:**

A systematic search of bibliographic databases for population-based and clinic-based studies of children aged 0–19, including all ethnic groups and the main child mental disorders. We synthesised findings by comparing each minority group to the White British study sample.

**Results:**

31 population-based and 18 clinic-based studies met the inclusion criteria. Children in the main minority groups have similar or better mental health than White British children for common disorders, but may have higher rates for some less common conditions. The causes of these differences are unclear. There may be unmet need for services among Pakistani and Bangladeshi children.

**Conclusion:**

Inter-ethnic differences exist but are largely unexplained. Future studies should address the challenges of cross-cultural psychiatry and investigate reasons for inter-ethnic differences.

## Background

Child mental health has deteriorated in Britain in the past 50 years [1,2], with the most recent and comprehensive estimates suggesting a 10% disorder prevalence [3,4]. Individual and family-level predictors of child mental health have been the focus of much research, but comparatively little is known about social and cultural variables such as ethnicity. That ethnic differences could have important public health implications is clear given ethnic diversity of children in Britain; in the 2001 census 85.7% of 0–19 year olds were White British, 2.2% White minority, 2.8% Mixed race, 1% Black Caribbean, 1.2% Black African, 2.2% Indian, 2.3% Pakistani, 0.9% Bangladeshi, and 1.7% of Chinese or other ethnicity [5]. Potential benefits of comparing health outcomes across ethnic groups include providing insights into aetiology, identifying health inequalities, and allowing meaningful interpretation of ethnic differences in service use [6]. This last point is of particular interest given growing policy emphasis upon making mental health services accessible and sensitive to children of all ethnicities [7].

There have been numerous discussions of ethnic differences in child mental health but this paper presents the first systematic review. It synthesises evidence from population- and clinic-based studies in Britain published over the last 40 years on the major child mental health problems in all minority ethnic groups.

## Methods

### Research questions

This review was motivated by two questions;

1. How, in population-based studies sampling from the general population, does the prevalence and proportional morbidity of mental health problems differ among children from different ethnic groups in Britain?

2. How do ethnic differences in levels and patterns of service use from clinic-based studies compare with estimates of disorder prevalence and proportional morbidity from population-based studies?

### Search strategy

We sought to identify all relevant quantitative studies produced at any time up to and including June 2007, following the guidelines of the expert working group consensus statement on the Meta-analysis of Observational Studies in Epidemiology (MOOSE) [8]. Between January and July 2007 we searched keywords, titles and abstracts in 16 electronic databases and eight websites [see Additional file 1]. Our search string combined a wide range of free text terms and subject index headings, and was evaluated and refined by assessing retrieval of known studies. Reference lists of articles considered for inclusion were scanned, as were previous discussions of the literature and non-systematic reviews [9-23]. Studies eligible for inclusion in this review were entered into the Science Citation Index to identify studies which had cited them.

To locate other relevant work, particularly unpublished studies, we asked for suggestions from experienced researchers in the field, circulated requests for assistance to five special interest groups [see Additional file 1], and contacted the corresponding authors of studies eligible for inclusion and published in the past 20 years. Finally, we sought to locate large epidemiological population-based studies of child mental health in Britain, because these seemed particularly likely to contain relevant information which would not necessarily be reported in an abstract. We located these studies through existing reviews [24-27] and through consulting other researchers.

### Inclusion criteria

The inclusion criteria were as follows:

• **Participants**: Living in Britain; aged 0–19 years; sampled from the general population or from mental health clinics serving the general population (i.e. not small and selected groups such as foster children or children in secure forensic units).

• **Ethnicity**: We operationalised ethnicity to include groups as defined by the 2001 UK Census [9]. Additional categories were added to cover groups whose religion, language or way of life serves in Britain as a marker for membership of a particular 'meaningful collectivity'. This included groups such as Orthodox Jews and Travellers but not, in the absence of additional information, internally diverse groups such as Christians or Muslims. Minority groups defined simply as 'minority', 'non-White' or 'other' were excluded. Included studies had to contain 1) at least two specified ethnic groups (not necessarily with one White/White British), or 2) one minority group compared to all other children in the sample/a comparable general population sample [see Additional file 1].

• **Mental health**: Included outcomes were: referral or admission to a child mental health service; "a psychiatric diagnosis" (unspecified) made by a mental health specialist; emotional disorders; behavioural disorders; hyperactivity disorders; less common disorders, including psychosis, autistic spectrum disorders and eating disorders; somatoform disorders; suicide and deliberate self-harm (DSH). Only validated clinical interviews or questionnaires were accepted, but validation in each ethnic group was not required. An experienced psychiatric epidemiologist judged whether enough evidence existed to establish the validity of interviews and questionnaires, doing so blind to study findings.

• **Study types**: Included study types were: 1) Population-based studies of prevalence or mean scores (minimum sample size N ≥ 40 for each included ethnic group for prevalence, N ≥ 10 for each included ethnic group for mean scores); 2) Clinic-based studies of the relative proportion of referrals/in-patients in clinics from ethnic minority groups, as judged against the ethnic composition of a base population such as the local catchment area (no minimum sample size); 3) Clinic-based studies which compared ethnic groups in terms of their proportional morbidity from different diagnoses – that is, the relative frequency of emotional disorders, of behavioural disorders etc among all mental health diagnoses. (minimum sample size N ≥ 20 for each included ethnic group).

• **Minimum sample sizes**: The minimum sample sizes described above were imposed to avoid highly underpowered studies leading to 'uninformative' null findings and/or publication bias. They varied for different study types depending on the estimated power to detect effects [see Additional file 1].

• No restriction was made on date or language of publication.

### Assessing studies for inclusion, data extraction and data analysis

All titles and abstracts (N = 6286) were assessed for possible relevance by the lead author (AG). A test-retest evaluation 4 weeks apart on 1391 of the electronically-retrieved studies demonstrated good reliability in this; AG re-identified 42 of the original 43 studies and no additional papers. Studies judged as potentially within the scope of the review were independently assessed for inclusion by AG and a second epidemiologist, with disagreement decided by consensus.

AG extracted data according to pre-determined fields for all mental health outcomes and all ethnic groups meeting our inclusion criteria, and judged studies against a pre-determined list of possible methodological limitations devised for the purposes of this review. These included limitations in the measurement of mental health; limitations in the measurement or reporting of ethnicity; methodological limitations which could cause selection or information bias; and the potential for confounding by age, sex and socio-economic position [see Additional file 1]. Data extraction and assessment of limitations were independently checked by a second epidemiologist, with the rare instances of disagreement decided by consensus. In some studies the relevant statistical tests were not reported but were 1) calculated by AG using data in the paper 2) calculated using data provided by the authors or 3) based on additional data analysis provided by the study authors. These calculations are indicated in the expanded results tables provided in Additional file 2.

We judged formal quantitative meta-analysis impossible because the classifications of ethnicity and of mental health outcomes were too heterogeneous. Instead we adopted a semi-quantitative descriptive approach which categorised the results of individual analyses according to whether each minority group considered showed evidence of more or fewer mental health problems than the White/White British/general population children in the study. Combined categories were used for studies showing discrepant findings for children from the same ethnic group according to different mental health outcomes, different informants or for different genders. In three studies [29-31], containing eight minority ethnic study populations, ANOVA analyses provided evidence (p < 0.05) of differences in mean scores, but post hoc contrasts between specific groups were not presented. These eight study populations could therefore only be tentatively grouped, based on the trend showed by the mean score in each ethnic group. Some studies presented not only the raw comparisons but also models which adjusted for a range of potential confounders. In such cases, we used the results of models adjusting only for age and gender or, failing that, we used the raw data/unadjusted models. This was done in accordance with our primary aim of *describing*, rather than explaining, ethnic differences.

## Results

### Description of studies

**Figure 1:**
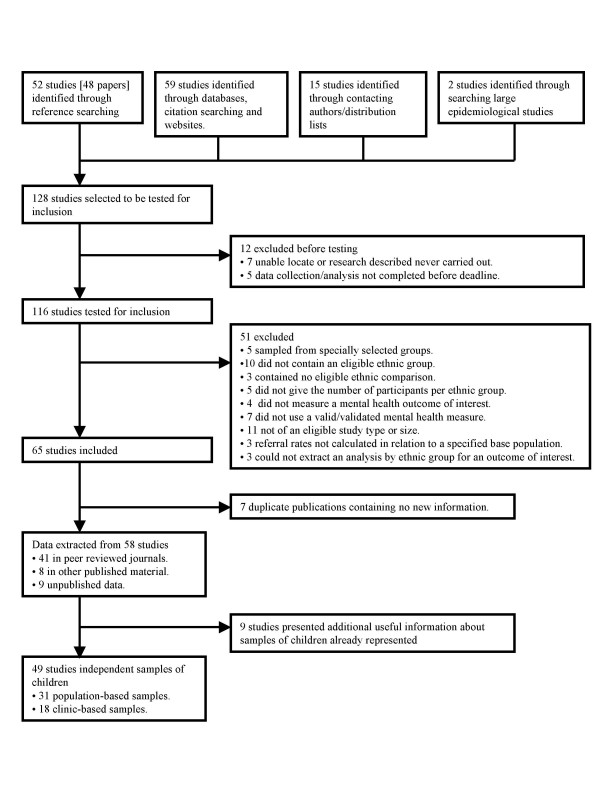
Selection of studies.

128 studies reported in 125 potentially relevant papers were identified, of which 116 studies had been completed and were successfully retrieved. As summarised in Figure 1, 58 of these studies were excluded [see Additional file 3] and 58 were included. The 58 included studies covered 49 independent samples of children, of which 31 were population-based [3,4,10-35] and 18 clinic-based [36-53]. Nine further population-based studies presented additional informative information on samples of children already represented [76-84]. All these included studies are described in detail in Additional file 2

Half of the included studies (25/49) have been published since 2000, reflecting the increasing interest in the mental health of children in minority ethnic groups. Most study populations were located in England (45/49 studies), particularly London (26/49 studies). Of the 31 population-based studies, 23 reported 'all disorders' or a common child mental disorder (emotional, behavioural or hyperactive); 7 reported disordered eating attitudes; and 1 reported psychotic-like experiences. Of the 18 clinic-based studies, 15 examined over- or underrepresentation of ethnic groups relative to the base population, and 7 examined proportional morbidity from different disorders. Of these 18 studies 13 reported 'all referrals/diagnoses' or a common child mental disorder; 5 reported psychosis; 7 reported deliberate self harm (DSH); and 3 reported other outcomes.

Although not specified in our inclusion criteria, all 49 studies included a White/White British/'general population' (i.e. largely White British) sample. This allowed us to have a single strategy for combining information across studies, by always comparing the results for each minority ethnic group to the White/White British sample. Of the minority groups listed in the UK census, only Black Caribbean, Indian and Bangladeshi children were included in ten or more studies, while White Minority and Chinese children feature in five or fewer.

The methodological limitations of individual studies are described in Additional file 2. The most common limitations related to the measurement or reporting of ethnicity (42/49 studies), the measurement or reporting of SEP (37/49), or potential selection bias through clinic-based sampling and/or low response rates (30/49).

### Population based studies of prevalence or mean scores

#### Common mental health problems

Table 1 summarises the results of population-based studies for the common child mental health problems. Black African and Indian children appear to enjoy better mental health than White British children, with at least one finding of an advantage reported in 5/6 studies of Black Africans and 8/12 studies of Indians. By contrast, most studies of Black Caribbean, Pakistani and Bangladeshi children found no evidence that their mental health differed from that of White British children. The mental health of Mixed race children also appears similar to that of White British children, although the diversity of this group complicates interpretation of this finding. Similarly the inconsistent findings from studies of 'Black' or 'South Asian' are hard to interpret given the potential heterogeneity of these ethnic categories. For other ethnic groups, including White minority and Chinese children, there is insufficient evidence to draw conclusions.

**Table 1:** Summary of findings of population-based studies of common child mental health problems/disorders

**Ethnic group**	**No. populations**	**Mental health problems/disorders relative to White/White British/'general population' children**				
		
		**Evidence of fewer problems**	**Mixture of evidence of fewer problems/no evidence of difference**	**No evidence of a difference**	**Mixture of evidence of more problems/no evidence of difference**	**Evidence of more problems**
**White Irish**	2	0	0	1	1	0
**White minority (unspecified)**	2	0	0	1	1	0
**Mixed race**	5	0	0	4 (?+1)	0	0
**Black Caribbean**	11	0	1	6	1 (?+1)	1 (?+1)
**Black African**	6 (in 5 papers)	3	2	1	0	0
**'Black'**	4	1	0	2	0	0 (?+1)
**Indian**	12	7 (?+1)	0	2	2	0
**Pakistani**	6	0 (?+1)	0	4	1	0
**Bangladeshi**	6	0	1	5	0	0
**'South Asian'**	5	1	1 (?+1)	1	0 (?+1)	0
**Chinese**	2	0	1	1	0	0
**Orthodox Jewish**	1	0	1	0	0	0

**Notes: **All differences significant at the 5% level, except for those shown in parentheses (e.g. '(?+1)') where the significance level for the specific contrast was not reported and where the study is therefore grouped by its apparent trend. Not all studies are independent, as some compare children from several minority ethnic groups to the same White 'reference' group.

For most ethnic groups, we have been unable to identify study characteristics which might explain discrepant findings between different studies. One important exception is Black Caribbean children, for whom teacher-reported findings of poor mental health in the 1970s have not been replicated more recently and by other informants. Evidence of poorer mental health comes predominantly from older studies (3/4 pre-1980 vs. 1/7 post-1980) and exclusively from teacher reports (4/5 teacher assessments across the 11 studies vs. 0/9 parent-, self- or multi-informant assessments). As most (3/4) pre-1980 studies used teacher-report while most (6/7) post-1980 studies did not, it is unclear how far this pattern reflects a post-1980 improvement in the mental health of Black Caribbean children and how far it reflects a tendency for teachers to make more negative assessments than other informants.

Seven studies, containing twelve study populations, distinguished emotional, behavioural and, in most cases, hyperactive problems. The five study populations of Indian children consistently indicate that where Indians had an overall advantage this was due to fewer behavioural/hyperactive disorders (3/3 studies [3,36,50]) and that where Indians showed an overall disadvantage this was driven solely by more emotional problems (2/2 studies [43,47]). The converse is suggested by four studies of Black Caribbean or Mixed White/Black Caribbean children [[33,54], 85], which found relatively more behavioural problems (3/4 study populations, although in one case in girls only [[33], 85]) and/or fewer emotional problems (2/3 study populations [54]). Finally the three study populations of Black African children reported fewer emotional and hyperactivity problems in Black Africans in two study populations [54] and fewer behavioural and, to a lesser extent, hyperactive symptoms in the third [35].

#### Eating disorders

Most of the evidence on problematic eating attitudes relates to South Asian girls. Four population-based studies reported higher questionnaire scores in South Asian girls [37,40,44] or, in one study, South Asian children (genders not disaggregated) [26]. Two further surveys showed marginal evidence of ethnic differences with the trend being towards higher questionnaire scores in South Asian girls [21] or South Asian children (genders not disaggregated) [27]. One final study showed no overall difference in South Asians girls compared to White British girls but an excess on one subscale [19]. Only one of these seven studies used clinician-based diagnoses in addition to questionnaire measures, with this study reporting some evidence of a higher prevalence in South Asian girls [17]. One study disaggregated 'South Asians', and reported that higher scores were confined to Bangladeshi girls, with Indians and Pakistanis scoring similarly to Whites [25].

Two of these surveys included minority ethnic groups other than South Asian. Both report no evidence of a difference relative to White children in Black children [26,27] but there is some evidence of an excess in the one, small Mixed race sample [26].

#### Psychosis

One population-based survey investigated psychotic-like experiences, reporting higher rates in Black Caribbean children, lower rates in South Asian/Chinese children, and no evidence of a difference for White minority or Black African children [55].

### Clinic-based studies of proportional representation of ethnic groups in clinic populations

Table 2 summarises the results of clinic-based studies of proportional representation of different ethnic groups for all disorders or all referrals. These studies compare the relative frequency of ethnic minority groups in the clinic with the ethnic composition of a base population such as the local catchment area. 'Overrepresentation' refers to instances in which the proportion of a particular minority ethnic group is higher in the clinic than in the base population, 'underrepresentation' refers to cases where the proportion is lower.

**Table 2:** Summary of findings of clinic-based studies of proportional representation among the clinic population

**Ethnic group**	**No. populations**	**Proportional representation of minority group relative to their share of the base population**		
		
		**Evidence of under-representation relative to the base population**	**Represented as expected relative to the base population**	**Evidence of over-representation relative to the base population**
**Black Caribbean**	1	0	1	0
**Black African**	1	0	1	0
**'Black'**	5	2	1	2
**Indian**	1	1	0	0
**Pakistani**	2	2	0	0
**Bangladeshi**	4	4	0	0
**'South Asian'**	6	3	2	1

**Notes**: All differences significant at the 5% level

There are seven small study populations of Black Caribbean, Black African or 'Black' children (N > 40 for expected referrals in only 1/7 populations). The findings are inconsistent and, in several cases, are very hard to interpret because of serious methodological limitations. These limitations include non-comparability of the ethnic classification system used for the clinic population and the base population in three studies [45,48,53], and the use of base population data 10 years out of date in a fourth [52]. By contrast, there is far more consistent evidence of underrepresentation of Indian, Pakistani, Bangladeshi and 'South Asian' children, this being seen in 10/13 study populations.

One clinic-based study of in-patients with psychosis found an overrepresentation of 'Black' children relative to the base population and no evidence of over- or underrepresentation in 'South Asian' children [52]. The authors comment that many of the Black in-patients with psychosis were refugees from Africa.

### Clinic-based studies of proportional morbidity of different disorder types

Six clinic-based studies examined proportional morbidity (i.e. the relative frequency of different disorders) for common mental health disorders. Two reported relatively fewer emotional disorders and relatively more behavioural disorders in Black Caribbean children [36,42], although in one study the excess of behavioural disorders was only seen in girls [36]. One study reported that Pakistani children presented with relatively fewer behavioural disorders and more 'adjustment' disorders [43]. Three reported no evidence that the relative frequency of problems differed in Bangladeshi [39,47] or South Asian children [40] compared with non-Bangladeshi/South Asian children.

Four clinic-based surveys included psychosis when investigating proportional morbidity among all referral reasons. One study investigated second generation Black Caribbean children and reports an excess of psychosis relative to other disorder diagnoses [42]. The remaining three studies reported no evidence of an excess in Bangladeshi [39,47] or 'South Asian' children [40], although the rarity of psychosis makes this a weak test for equality.

Five clinic-based studies specifically investigated deliberate self harm (DSH). These provide no evidence of a difference, relative to White British children, in Black Caribbean children [37,44] or South Asian children aged under 15 [38,41,49]. One study does, however, report that South Asians females (but not males) aged 15–19 were overrepresented [38]. Two further clinic-based studies of 'all referrals' in Bangladeshi children found no proportional excess of DSH [39,47], although the small number of referrals for DSH makes this a weak test for equality.

One proportional morbidity analysis reported a relative overrepresentation of 'adjustment disorders' in Pakistani children [43], and another that somatoform disorders were relatively more common in 'South Asian' boys [40]. A final study of proportional morbidity found a relative excess of autistic spectrum disorders in Black Caribbean children [42].

## Discussion

### Limitations of the review

Before discussing our findings, it is worth highlighting some limitations of this review. Publication bias is particularly acute for routinely collected variables like ethnicity, as is the problem of relevant findings being 'hidden' in the main body of reports but not included in the abstract. Despite our multiple-pronged approach, we are therefore likely to have missed some studies, particularly those reporting null findings.

The heterogeneity of exposures and outcomes in this review made formal meta-analysis techniques impossible. We grouped studies by whether they reported statistically significant differences at the 5% level between minority groups and White/White British children because we felt this approach helped to clarify trends in the data. This method does, however, have several major limitations, including giving inadequate weight to studies reporting large and highly significant effects and giving too much weight to underpowered studies reporting 'no effect'. In addition, to avoid favouring studies including multiple testing on the same subjects, we presented each study only once, using combined categories such as 'non-significant/better mental health' where necessary. This does, however, give insufficient weight to studies showing consistent findings across multiple informants or by multiple measures. Like most meta-analyses, we also synthesised evidence without regard to variation in study quality.

A further, major drawback of the method we use is that it reinforces the idea that White/White British children represent an invariant, normative benchmark. This obscures important potential differences within this group children both by ethnicity (e.g. migrants from different European countries) and by other characteristics such as geographic region or socio-economic position. We hope to have reduced the problem of geographic and socio-economic variation somewhat, however, by basing our analysis upon ethnic comparisons *within *studies or with comparable general population samples.

### Findings for common mental disorders

For common disorders, population-based studies suggest that Black African and Indian children may enjoy better mental health than White British children, while the mental health of Mixed race, Black Caribbean, Pakistani and Bangladeshi children is similar. For other minority groups there is insufficient evidence to make any evaluation.

The causes of these of inter-ethnic similarities and differences have been little investigated and remain largely unexplained. Only eight population-based studies of common mental disorders examined possible mediating or confounding factors. These include three large, recent studies which adjusted for a number of measures including individual child factors family structure, family social support, family activities, various measures of socio-economic position (SEP) and area deprivation [3,54,56]. This adjustment had little effect on observed advantages and unmasked a relative advantage in other minority groups (namely Pakistani [3], 'Black'[56] and Black Caribbean [54]). The one case where an advantage in univariate analysis disappeared upon adjustment is plausibly because the advantaged group 'Indian' was collapsed into a broader 'South Asian' group [57]. Instances of disadvantage in minority ethnic groups have also been relatively little investigated, but may in some cases be partly explained by lower SEP [15], social support [28] and migration-related factors [10,29].

Within the common mental disorders, Indian children seem to display relatively more emotional and/or fewer behavioural problems, while the converse may be true of Black Caribbean and Mixed White/Black Caribbean children. Relatively fewer emotional and/or more behavioural problems were also observed in the two clinic-based studies of proportional morbidity of Black Caribbean children.

### Findings for less common disorders and deliberate self-harm

There is a reasonably consistent finding that South Asian girls (or, in some studies, South Asian children) receive higher scores than White British girls on eating disorder questionnaires. Interpreting these ethnic differences is, however, complicated by the possibility that the meaning attributed to these questionnaires may differ among British South Asians. There is some evidence suggesting that this is the case for girls living in the Indian subcontinent [58], and circumstantial evidence that a similar phenomenon could exist in Britain is suggested by the three studies which identify possible mediating factors. In all three cases, the mediating factors are ones which could plausibly be associated with attitudes and cultural values more similar to those in the Indian subcontinent (parental 'overprotection' in two studies [19,20] and a 'traditional' orientation in the child in a third [17]). One research priority is therefore to investigate whether these questionnaire differences are replicated in studies using diagnostic interviews or clinical diagnoses.

For psychosis and DSH, our review hints at interesting continuities and discontinuities between childhood and adulthood. Three studies (one population- and two clinic-based) investigate psychosis or psychotic-like experiences in Black Caribbean, Black African or 'Black' children. Both Black Caribbean samples and the one 'Black' sample are at higher risk, which mirrors the findings in adults [59,60], although in the one Black African sample there is no difference. Conversely, given some evidence of elevated DSH among young South Asian women [61], the *absence *of excess risk of DSH in South Asians aged under 16 is noteworthy. These observations are based on only three studies each, five of which are clinic-based (therefore carrying a greater risk of selection bias through differential referral) and five of which use the unsatisfactory groupings 'Black' and 'South Asian'. We hope, however, that these preliminary observations may motivate further research into when and how these mental health problems develop in the transition between childhood and adulthood.

### Representation of ethnic groups in mental health services

The underrepresentation of minority groups in mental health clinics has attracted considerable attention and is typically explained in terms of unmet need [62]. This review clarifies the evidence on this point and, by comparing clinic-based and population-based evidence, can shed some light on the question of unmet need. For Black Caribbean, Black African or 'Black' children evidence of an underrepresentation is sparse, inconsistent and based on studies with important methodological limitations. By contrast, for Indian, Pakistani, Bangladeshi and 'South Asian' children there is a larger and more consistent body of evidence supporting underrepresentation in clinic populations relative to their share of the local/catchment population.

Given that this review defined under- or over-representation relative to each ethnic group's share of the local base population, it is important to stress that underrepresentation does not necessarily imply inequity in access to health services. On the contrary, lower service use may be appropriate for Indians, whom population-based studies suggest enjoy better mental health. The degree of underrepresentation in clinic populations seen in Pakistanis and Bangladeshis is, however, considerably larger than any differences seen in population-based studies. This supports the explanation of greater unmet need in Pakistanis and Bangladeshis as, circumstantially, does the fact that the one clinic where South Asians were *over*represented had recently invested heavily in a South Asian Outreach Project [53]. Any decision to target particular ethnic groups must, however, be preceded by careful examination of the source of unmet need; for example culturally inappropriate services vs. low rates of recognition in the community. A decision to target should also be balanced against the evidence of large unmet need in children of all ethnicities [63].

### Priorities for future research

This review reveals heterogeneity in the mental health of children from different ethnic groups, including within the groups 'Black' and 'South Asian'. It therefore underlines the importance of defining and reporting ethnicity in at least as much detail as the UK census 2001 [9]. This review also highlights the difficulty of interpreting studies in which ethnicity is defined in the clinic population using a different classification system to that used in the base population (e.g. one without the category 'Mixed race'). Fortunately, the widespread adoption of the census 2001 classification within the NHS should ameliorate both these issues.

If improved measurement of ethnicity is important, so too is more sophisticated evaluation of mental health outcomes. Twenty-two of the 31 population-based studies in this review rely exclusively on brief mental health questionnaires, which may yield misleading findings if there are inter-ethnic differences in the experience, perception or reporting of symptoms. A more rigorous and more systematic approach to addressing the challenges of cross-cultural research is needed through strategies such as using detailed interview-based measures in addition to questionnaires; examining the internal consistency of questionnaire subscales; comparing inter-informant agreement; and including a qualitative component to research projects.

Beyond these issues of measurement, future research should also pay more attention to issues of explanation. Ethnicity is multi-faced construct which combines biological elements, ethnic self-identification with a 'meaningful collectivity', and the broader social and institutional factors shaping the experiences of particular groups [64]. Precisely for this reason, the observation of any particular pattern of inter-ethnic similarities and differences should be a starting point for further hypothesis-driven investigations of causal mechanisms. These investigations must consider the possibility of inter-ethnic biases in the measurement of mental health problems in different groups, including issues of language or differential psychometric properties of measurement tools. They should then use qualitative and/or quantitative approaches to investigate directly which child, family, community or area characteristics may underlie any observed differences which do appear genuine. Examining whether ethnic effects vary across different groups of children (e.g. different ages or genders) is also of interest and may help focus research on plausible causal mechanisms. Disappointingly few studies rose to these central challenges, and as a result the causes of the apparent inter-ethnic differences observed in this review remain unclear.

## Conclusion

In summary, the prevalence of common mental health problems in the main minority ethnic groups in Britain seems to be similar to or, in some specific minorities, lower than that of White British children. This absence of evidence for a *dis*advantage is certainly reassuring, and is also striking given the socio-economic adversity which many minority groups face [65]. Yet equality with White British children still corresponds to a high burden of mental disorders (approximately 10% prevalence). Moreover, for several small minority groups there is simply insufficient evidence to make any meaningful evaluation for the common mental disorders. There is also some suggestion, albeit based on inconclusive evidence, of more psychosis in Black children, more disordered eating attitudes in South Asians, and greater unmet need for mental health services in Pakistani and Bangladeshi children.

The aetiology of these observed inter-ethnic similarities and differences is unclear, but potentially of great interest. In particular, understanding the apparent advantage in some groups, and the absence of a disadvantage in other groups despite socio-economic adversity, could yield important insights into protective factors against child mental health problems. The vital next steps are therefore to address more fully the challenges of cross-cultural psychiatric research, including the possibility of cross-cultural biases; and then to use qualitative and/or quantitative research to investigate directly possible causal mechanisms for any genuine differences. This is crucial for understanding how and why mental health varies across ethnic groups; an understanding which could hold important clues to improving the well-being of children of all ethnicities.

## Competing interests

The authors declare that they have no competing interests.

## Authors' contributions

AG participated in defining the scope and purpose of the review, located and processed studies, synthesised the findings, and drafted the manuscript. VP and DAL participated in deciding the aims and methods of the review, interpreting the results, and helped to draft the manuscript. All authors read and approved the final manuscript.

## Pre-publication history

The pre-publication history for this paper can be accessed here:



## Supplementary Material

Additional file 1 **Supplementary information on methods**. Supplementary information on judging comparability of general population samples to study samples, calculation of minimum sample sizes, and details of methodological limitations. Click here for file

Additional file 2 **Full description of included studies**. Detailed description of individual studies included in the review. Click here for file

Additional file 3 **Full description of excluded studies**. References for excluded studies, with a more detailed description of why they were excluded: expands on the information in Figure 1. Click here for file
