# Supplementary material for: Child mental health differences amongst ethnic groups in Britain: a systematic review
Source: BMC Public Health. 2008 Jul 25;8:258. doi: 10.1186/1471-2458-8-258 (PMC2515844; doi:10.1186/1471-2458-8-258)
Supplement: Additional file 1 — Supplementary information on methods. Supplementary information on judging comparability of general population samples to study samples, calculation of minimum sample sizes, and details of methodological limitations. [file 1471-2458-8-258-S1.doc]

# Additional file 1 – Supplementary information on methods

## Details of electronic search strategy

## *Search string*

(#Infant OR #Child OR #Adolescent) AND (#Mental health OR #Mental disorder OR #Psychiatry OR #Psychiatric clinics OR “mental health” OR “mental illness” OR “mental distress” OR “mental disorder*” OR “behaviour disorder*” OR “behavior disorder*” OR “emotional disorder*” OR “hyperactiv*” OR “hyperkinesis” OR externali*ing OR internali*ing OR anorexia OR bulimia OR “eating disorder*” OR self-harm OR self-injur* OR suicid* OR somatoform OR autism OR autistic OR psychosis OR psychotic OR psychoses) AND (#Britain OR #United Kingdom OR “British” OR “Britain”) AND (#Ethnic groups OR #Ethnic differences OR #Minority groups OR#Cross-cultural comparison OR Ethnic* OR Migrant* OR Immigrant* OR Minorit* OR Race OR Racial OR “cross-cultural” OR “cross cultural” OR White OR Caucasian OR mixed race OR Black OR African OR Afro OR West Indian OR Asian OR Indian OR Bangladeshi OR Bengali OR Pakistani OR Punjabi OR Gujarati OR Tamil OR Chinese)

# = indicates exploded indexing terms to include all subheadings. * = wildcard symbol. Search terms in quotes searched as exact phrases

## *Databases*

- BNI*
- British Library theses index
- CAB Direct
- CINAHL
- Cochrane
- DH National Research Register*
- DH ReFeR*
- ESTAR
- Embase
- HMIC
- IBSS
- PubMed
- PsycINFO
- Science and Social Science Citation Index
- TRIP
- Zetoc

* ‘Britain’-related terms omitted from search strings

## *Websites*

- Centre for Evidence in Ethnicity, Health and Diversity (http://www2.warwick.ac.uk/fac/med/research/csri/ethnicityhealth/)
- Centre for Research in Ethnic Relations (http://www2.warwick.ac.uk/fac/soc/crer)
- The NHS Specialist Library for Ethnicity and Health (www.library.nhs.uk/ethnicity)
- Confederation of Indian organisations (http://www.cio.org.uk/)
- The Department of Health National electronic library for health (http://www.kingsfund.org.uk/)
- The King’s Fund (http://www.kingsfund.org.uk/)
- Mind (National Association for Mental Health) (http://www.mind.org.uk/Information/Factsheets)
- The National Ethnic Minority Data Archive (http://www.warwick.ac.uk/~errac/nempubs.htm)

## *Special interest groups and e-mail distribution lists*

- The Royal College of Psychiatry’s Transcultural Psychiatry special Interest Group.
- The Royal College of Psychiatry’s faculty of Child and Adolescent Psychiatry.
- CAMHS@JISCMAIL.AC.UK
- YOUNGPERSONS-PSYCHIATRIC-NURSING@WWW.JISCMAIL.AC.UK
- MINORITY-ETHNIC-HEALTH@JISCMAIL.AC.UK

## Criteria for the a general populations sample to be considered a comparable group

Criteria for the general population sample being considered comparable were: matched for population vs. clinic-based; nationally representative sampling or representative sampling from the same geographic area (operationalised as a Government Office Region); same mental health outcome(s) and measures; and matched for year of data collection to ±10 years.

## Calculation of minimum sample sizes

The minimum size restriction was introduced because null findings involving very small numbers become ‘uninformative’, being better interpreted as an absence of evidence rather than evidence of an absence. Moreover, precisely because a null finding based on so few individuals is so uninformative, such findings are unlikely to be published and publication bias is likely to become particularly acute. Our minimum ethnic group sizes are based on power calculations with significance at 5% and power set at 50% - i.e. the level at which half of genuine differences will be missed. This, in combination with the assumption of relatively large ethnic differences, deliberately sets the bar for inclusion quite low. The power calculations also make allowance for the possibility that a minority group may only make up a small proportion of the total sample population.

*Minimum group size for population-based prevalence or mean score studies*

- N≥40 for prevalence for each included ethnic group, corresponding to a difference of 10 vs. 25% (for questionnaire cut-offs) or 10% vs. 3% (for disorder prevalences).
- N≥10 for mean scores for each included ethnic group, based on effect size of 0.7.

*Minimum group size for clinic-based studies of the relative proportion of referrals/in-patients in clinics from ethnic minority groups*

- No minimum group size.

*Minimum group size for clinic-based studies of the proportional morbidity of different disorders*

- Minimum group size N≥20 for each included ethnic group, based on moving from a 50:50 split of e.g. internalising and externalising disorders to a 75:25 split.

## Full details of methodological limitations

*Measure of mental health*

- **A:** Mental health information only from a questionnaire measure.
- **B:** Reliance on a single, inappropriate informant. Inappropriate informants were defined as a) self-report by child aged under 11, b) self-report where oppositionality and hyperactivity are the key outcomes (anti-social behaviour scales accepted), or c) teacher-report where emotional disorders, eating disorders or deliberate self-harm are the key outcomes.
- **C:** Non-validated modification of a validated mental health score.

*Measurement of ethnicity*

- **D:** Method of assigning ethnicity not described.
- **E:** Ethnicity determined by a potentially inferior method. Adequate methods were child’s self report, parent’s report on child, or according to parent’s /grandparent’s country of birth/’country of origin. Other methods were considered inferior - for example using *child’s* place of birth, ethnicity as assigned by name, or ethnicity as ascribed by clinicians. Where case notes were used to assign ethnicity, but it was not explicitly stated how information on case notes was completed, this was recorded ?E.
- **F:** Ethnicity analysed using meta-level descriptions only (‘White’ (combining White British/Irish with White minority), ‘Mixed’, ‘Black’ or ‘Asian’/‘South Asian’) or by comparing one ethnic group to a potentially mixed-ethnicity comparison group (e.g. all other children in the sample/the ‘general population’).

*Methodological limitations of the study that may cause bias*

- **G:** Potential for selection bias - clinic-based sampling.
- **H:** Potential for selection bias - response rates less than 60% for population-based surveys **or** completeness of ethnicity data less than 60% for clinic surveys.
- **I:** Potential for information bias - investigator-based ratings made by the study authors without being blinded to ethnicity.

*Alternative explanations for observed differences*

- **J:** Differences could be due to confounding by age and sex (including cases where no information was given). This was taken to apply *unless* a) study was restricted to one sex or age range ≤ 3 years b) similar age (< 1 year difference) and/or sex profile (<10% difference) was demonstrated in the different ethnic groups, c) results were stratified by age (age bands ≤ 3 years) and/or sex, or d) age and/or sex were controlled for in multivariate analyses. **This criterion was NOT applied to clinic based referral rate studies** – i.e. it was assumed that the *base* population was balanced in its age and sex composition.
- **K:** No data presented on socio-economic position
- **L:** No adjustment made for reported differences in socio-economic position.
