# Supplementary material for: Child mental health differences amongst ethnic groups in Britain: a systematic review
Source: BMC Public Health. 2008 Jul 25;8:258. doi: 10.1186/1471-2458-8-258 (PMC2515844; doi:10.1186/1471-2458-8-258)
Supplement: Additional file 2 — Full description of included studies. Detailed description of individual studies included in the review. [file 1471-2458-8-258-S2.doc]

# Additional file 2 – Full details of included studies

## Table 4: Detailed description of population-based studies

| **Study ref.** | **Pub. type** | **Setting, date** | **Study design: study population** | **Ethnicity assignment** | **Mental health outcome: informant(s)** | **Results** | **Limitations** |
| --- | --- | --- | --- | --- | --- | --- | --- |
| **Bagley 1972 [1]** | 1 | London, ?? | Cross-sectional: 186 children aged 7: 112 White British/Irish; 74 Black Caribbean | Not specified | Common mental problems: teacher | Black Caribbean ↑ | A, D, F, ?H, ?J (sex), K |
| **Rutter 1974 [2]; 1975 [3]** | 1; 1 | London, 1970 | 2-stage cross-sectional: 2 043 children aged 10: First stage: 1689 White; 354 Black Caribbean. Second stage: 265 White; 118 Black Caribbean | Country of origin | Common mental problems/disorders: teacher and (second stage) parent | Black Caribbean ↑/— | F, L |
| **Kallarackal 1976 [4]** | 2 | Leicester, ?? | Cross-sectional: 198 children aged 9-12: 98 White British; 100 Indian | Not specified | Common mental problems: parent and teacher | Indian ↓* | A, D, L |
| **Cochrane 1979 [5]** | 1 | Birmingham, 1976 | Cross-sectional: 301 children aged 9: 74 White British; 87 Black Caribbean; 42 Pakistani; 98 Indian | Country of origin | Common mental problems: teacher | Black Caribbean ?↑; Pakistani ?↓; Indian ?↓ | A |
| **Earls 1980 [6]** | 1 | London, 1972-73 | Cross-sectional: N=763 children aged 3: 705 White British; 58 Black Caribbean | Country of origin | Common mental problems: parent | Black Caribbean —* | A, I, L |
| **Osborn 1985 [7]** | 2 | Great Britain, 1975 | Prospective cohort study 12 335 children aged 5: 11907 White British; 187 Black (>90% Black Caribbean); 241 South Asian | Country of origin | Common mental problems: parent | Black ?↑; South Asian ?↑/— | A, F, H (for black children) |
| **Hackett 1991 [8]** | 1 | Manchester, ?? | Cross-sectional: 200 children aged 4-7: 100 White British;100 Gujarati Indian | Not specified | Common mental problems/referral: parent | Indian ↓* | D, H, ?J (sex), L |
| **Mumford 1991 [9]** | 1 | Bradford, ?? | 2-stage Cross-sectional: 559 girls aged 14-15: 255 White; 204 South Asian | Not specified | Problematic eating attitudes/eating disorder: self-report and clinical diagnosis | South Asian ↑ | D, F, I, K |
| **Newth 1993 [10]** | 1 | Birmingham, 1987 | Cross-sectional: 194 children aged 3-4: 65 White; 129 South Asian (111 Pakistanis; 18 Indians) | Not specified | Behavioural problems/disorders: parent and clinical rating | South Asian ↓/—* | ?E, F, I, ?J (sex), L |
| **Ahmad 1994 [11]** | 1 | Bolton, 1993† | Cross-sectional: 186 girls aged 14-15: 115 White British; 71 South Asian | Reported by child | Problematic eating attitudes: self-report | South Asian — | A, F, K |
| **McCourt 1995 [12]** | 1 | Birmingham, ?? | Cross-sectional: 336 girls aged 12-16: 158 White; 178 South Asian | Not specified | Problematic eating attitudes: self-report | South Asian ↑ | A, D, F, K |
| **Waller 1995 [13]** | 1 | Birmingham, ?? | Cross-sectional: 260 girls aged 14-15: 107 White; 153 South Asian | Not specified | Problematic eating attitudes: self-report | South Asian — | A, C, D, F, K |
| **Marks 1997 [14]** | 1 | London, ?? | Cross-sectional: 174 children aged 5-11: 61 White British; 113 Bangladeshi | Not specified | Common mental problems/disorders: teacher and clinical rating | Bangladeshi —* | B, D, ?H ?I, K |
| **Nikapota 1998 [15]** | 3 | London, 1996-97 | Cross-sectional: 258 children aged 9-12: 60 White British; 40 Mixed White-Black Caribbean; 60 Black Caribbean;[38 Black African]; 60 South Asian | Reported by child's parent | Common mental problems/disorders: parent and teacher | Mixed race ?—; Black Caribbean ?↑/—; South Asian ?↓/— | F, ?H, ?I, J (sex), L |
| **Nazroo 1999 [16]** | 2 | England, 1999 | Cross-sectional: 1914 children aged 4-15 : 342 Irish; 363 Black Caribbean; 296 Indian; 412 Pakistani; 319 Bangladeshi; 182 Chinese. Compared with a 1997 general population sample of 5705 children. | Reported by child/child's parent | 1) Common mental problems: parent. 2) emotional problems (for those aged 13-15): self-report | White Irish ↑/—; Black Caribbean —; Indian ↑/—; Pakistani ↑/—; Bangladeshi —; Chinese —. | A, F, J (age), K |
| **Meltzer 2000 [17]; Ford 2003 [18]; Ford 2004 [19]; Evans 2004 [20]** | 2; 1; 1; 1 | Great Britain, 1999 | Cross-sectional: 10 438 children aged 5-15: 9529 White; 105 Black-Caribbean; 74 Black African; 222 Indian; 147 Pakistani; 43 Bangladeshi; [34 Chinese; 284 Other] | Reported by child's parent | Common mental disorders: clinical rating. | Black Caribbean—**; Black African —**; Indian ↓**; Pakistani —**; Bangladeshi —** | F |
| **Furnham 2001 [21]** | 1 | London, 1998 | Cross-sectional: 168 girls aged 15-17: 46 White; 40 Indian; 44 Pakistani; 38 Bangladeshi | Country of origin | Problematic eating attitudes: self-report | South Asian ↑ (Indian ?—; Pakistani ?—; Bangladeshi ?↑) | A, ?F, K |
| **Thomas 2002 [22]** | 1 | Bristol, ?? | Cross-sectional: 653 children aged 11-16: 405 White; 19 Mixed race; 101 Black; 103 South Asian; [25 Other] | Reported by child | Problematic eating attitudes: self-report | Mixed race ↑; Black —; South Asian ↑ | A, F, ?H, J (age), K |
| **Bhugra, Bhui 2003 [23]** | 1 | London, ?? | Cross-sectional: 266 children aged 13 or more: 134 White British; 51 Black Caribbean; 52 South Asian; [29 Other] | Reported by child | Problematic eating attitudes: self-report | Black —; South Asian — | A, C, F, ?H, K |
| **Atzaba-Poira 2004 [24]; 2005 [25]; In Press [26]** | 1; 1; 3 | London, 2001-02; 2005-06 | Repeat cross-sectional: Time 1: 125 children aged 7-9: 59 White British; 66 Indian. Time 2: 68 children aged 9-13 (58 from Time 1): 37 White British; 31 Indian | Country of origin | Common mental problems: parent and, in time 1 only, teacher | Time 1: Indian —/↑ Time 2: Indian —/↑ | A, ?H |
| **Stansfeld 2004 [27]; Klineberg 2006 [28]; Fagg 2006 [29]** | 1; 1; 1 | London, 2001 | Cross-sectional: 2790 children aged 11-14: 581 White British; 161 White minority; 194 Mixed race; 575 Black; 250 Indian; 184 Pakistani; 690 Bangladeshi; [115 Other] | Reported by child | 1) Common mental problems and 2) emotional problems: self-report | White minority — /↑; Mixed race —; Black —; Indian —; Pakistani —; Bangladeshi ↓/— | A, F (for Fagg 2004) |
| **Edmunds 2005 [30]** | 1 | London, 2003 | Cross-sectional: 163 children aged 5: 78 White; 18 Mixed; 34 Black; 33 South Asian | Reported by child's parent | Common mental problems: parent | Mixed —***; Black —***; South Asian —*** | A, F, H |
| **Flouri 2005 [31]** | 1 | South England, 2001-02 | Cross-sectional: 582 children aged 11-19: 360 White British; 222 Indian | Reported by child | Common mental problems: self-report | Indian ↓ | A |
| **Frosh 2005 [32]** | 1 | London, ?? | Cross-sectional: 341 children aged 5-15: Parent report: 161 Orthodox Jews; 10 298 children from a nationally representative general population sample collected in 1999. Teacher report: 325 Orthodox Jews, 8 028 general population. | Other method | Common mental problems: parent and teacher | Orthodox Jew ↓/—* | A, E, F, H (for parents), J (age), L |
| **Green 2005 [33]** | 2 | Great Britain, 2004 | Cross-sectional: 7 974 children aged 5-16: 6787 White British; 134 White minority; 223 Mixed; 91 Black-Caribbean; 87 Black African; 197 Indian; 229 Pakistani; 79 Bangladeshi; [16 Chinese, 131 Other] | Reported by child's parent | Common mental problems: clinical rating. | White minority —**; Mixed race —**; Black Caribbean —**, Black African ↓**; Indian ↓**; Pakistani —**; Bangladeshi —** | F |
| **Berry 2006 [34]** | 2 | Birmingham and Leicester†, ?? | Cross-sectional: 240 children aged 13-18: 120 White British; 120 Indian | Reported by child | Behavioural problems: self-report | Indian ↓* | A, C, ?H, |
| **Fuller 2006 [35]** | 2 | England, 2004 | Cross-sectional: 1120 children aged 4-15: 212 Irish; 168 Black Caribbean; 148 Black African; 194 Indian; 185 Pakistani; 116 Bangladeshi; 97 Chinese. Compared with a nationally representative general population of 5882 children from 2002 | Reported by child's parent | Common mental problems: parent | White Irish —; Black Caribbean —; Black African ↓/—; Indian —; Pakistani —; Bangladeshi —; Chinese —/↓. | A, F, J (age), K |
| **Scott 2006 [36]** | 2 | London, 2001-04 | Cross-sectional: 174 children aged 4-7: 42 White British; 32 Black Caribbean; 79 Black African; [21 Other] | Reported by child's parent | Common mental problems (various measures): parent, teacher, directly observed behaviour | Black Caribbean —/↓**; Black African —/↓** | I, K |
| **Wardle 2006 [37]** | 1 | London, 1999-2004 | Prospective cohort study N=5704 children aged 11/12 at baseline and followed up to age 14/15: Over the full study period: 3324 White; 1482 Black/mixed black (1289 Black, 193 mixed black); 627 South Asian/mixed Asian (557 Asian, 70 mixed Asian); [271 Other] | Reported by child | Common mental problems: self-report | Black/mixed Black ↓***; South Asian/Mixed Asian ↓*** | A, F, L |
| **Laurens 2007 [38]** | 3 | London, ?? | Cross-sectional: 595 children aged 9-12: 200 White British; 45 White Minority; 102 Black Caribbean; 155 Black African; 45 South Asian/Chinese; [43 Other] | Reported by child's parent | Psychotic-like experiences: self-report | White minority —; Black Caribbean ↑; Black African —; South Asian/Chinese ↓ | A, ?C, F, H, K |
| **Maynard submitted [39]; Maynard 2007 [40]** | 3; 1 | London, 2003† | Cross-sectional: 4635 children aged 11-13: 1224 White British; 297 Mixed White British/Black Caribbean; 926 Black Caribbean; 609 Nigerian or Ghanaian; 464 Other African; 494 Indian; 621 Pakistani/Bangladeshi | Reported by child | Common mental problems: self-report | Mixed Black Caribbean/White —; Black Caribbean —; Nigerian/Ghanaian ↓; Other African ↓; Indian ↓ | A |
| **Notes to Table 4 and 5**: CAMHS = Child and Adolescent Mental Health Services. † = information provided through personal communication with authors. **Publication type**: (1)=published in peer-reviewed journal, (2)=published outside a peer-review journal (e.g. book), (3)=unpublished report/under submission. Ethnic groups listed in square brackets are not presented in results because of insufficient numbers or because they were of ‘Other’ ethnicity and therefore ineligible. **Results**: ↑ = evidence at the 5% significance level of more mental health problems relative to White/White British/general population, ↑/— = mixture of evidence of more mental health problems and no difference; — = no evidence of difference; ↓/— = mixture of evidence of fewer mental health problems and no difference; ↓ = evidence of fewer mental health problems. '?' indicates the apparent trend in cases where there was evidence of overall ethnic differences, but post hoc tests of specific contrasts were not provided. * = statistical calculations performed by AG using information in the text ** = statistical calculations performed by AG using information supplied separately by the authors. ***=unpublished results obtained by AG from authors. **Methodological limitations** (full details in Additional file 1): A = Questionnaire measure of mental health only. B = Reliance on a single, inappropriate informant C = Non-validated modification of a validated mental health measure. D = Method of assigning ethnicity not described. E = Ethnicity determined by an inferior means. F Ethnicity analysed using meta-level descriptions (e.g. ‘South Asian’) G Potential for selection bias - clinic-based sampling. H Potential for selection bias - response rates/data completeness <60%. I Potential for information bias - investigator-based ratings made by the study authors without being blinded to ethnicity.. J Differences could be due to confounding by age and sex. K No data presented on socio-economic position. L No adjustment made for reported differences in socio-economic position. '?' indicates insufficient information to know whether a limitation applied. | | | | | | | |

## Table 5: Detailed description of clinic-based studies

| **Study ref.** | **Publi-cation** | **Setting, date** | **Study design: study population** | **Ethnicity assignment** | **Mental health outcome: informant(s)** | **Results** | **Limitations** |
| --- | --- | --- | --- | --- | --- | --- | --- |
| **Graham 1967 [41]** | 1 | London, 1963-65 | Retrospective case notes analysis: 110 children: 55 White British; 55 Black Caribbean | Not specified | Proportional morbidity for diagnosis | Black Caribbean PM: behavioural —/↑; emotional ↓ mixed emotional/behavioural — | ?E, G, L |
| **Taylor 1984 [42]** | 1 | London, ?? | Case-control study 100 children aged 8-17: 14 Black Caribbean; 86 non-Black Caribbean | Country of origin | Proportional morbidity for Deliberate self harm | Black Caribbean PM: DSH — | F, G, K |
| **Glover 1989 [43]** | 1 | London, 1980-84 | Retrospective case notes analysis: 156 girls aged 15-19: 25 South Asian; 131 non-South Asian | Not specified | Deliberate self harm | South Asian ↑/— | D, F, G, ?H, K |
| **Stern 1990 [44]** | 1 | London, 1987 | Retrospective case notes analysis.: 189 children (142 from Tower Hamlets): Tower Hamlets referrals: 18 Bangladeshi; 124 non-Bangladeshi. All referrals: 27 South Asian; 162 non-South Asian | Not specified | 1) Referral to CAMHS 2) Proportional morbidity for referral reason | Tower Hamlets Bangladeshi ↓*. All Bangladeshi PM; Behavioural —; emotional —; DSH —; psychosis — | ?E, F, G, J (age, sex), K |
| **Jawed 1991 [45]** | 1 | Bolton, 1981-85 | Retrospective case notes analysis.: 978 children: Referral data for 45 South Asian, 933 non-South Asian (although it is unclear if details were simply unrecorded in some cases). Diagnosis information on 72 White British; 45 South Asian | Not specified | 1) Referral to CAMHS 2) Proportional morbidity for diagnosis | South Asian ↓*. South Asian PM: behavioural ↓/—; emotional —; hyperactivity —; somatoform ↑/—; psychosis — | ?E, F, G, ?H, J (age), K |
| **McGibben 1992 [46]** | 1 | Coventry, 1982-90 | Retrospective case notes analysis: 340 children aged 12-15: 295 White; 45 South Asian | Not specified | Deliberate self harm | South Asian —* | ?E, F, G, ?H, K |
| **Goodman 1995 [47]** | 1 | London, 1973-89 | Retrospective case notes analysis: 1603 children aged under 18: 292 second generation Black-Caribbean; 1311 comparison group with both parents born in Britain. | Country of origin | Proportional morbidity for diagnosis | Black Caribbean PM: behavioural ↑; emotional ↓; mixed emotional/behavioural —; hyperactivity —; autism ↑; psychosis ↑ | F, G |
| **Roberts 1995 [48]** | 1 | Bradford, 1987-91 | Retrospective case notes analysis.: 2 462 children aged 2-17: Referrals: 184 Punjabi Pakistani Muslims; 2 278 non-Punjabi Pakistani. PM for diagnoses: 184 White British; 184 Punjabi Pakistani | Not specified | 1) Referral to CAMHS 2) Proportional morbidity for diagnosis | Pakistani ↓. Pakistani PM: behavioural ↓; anxiety —; adjustment disorders ↑ | ?E, G, ?H |
| **Goddard 1996 [49]** | 1 | London, 1990-92 | Retrospective case notes analysis: 100 children aged 10-17 : 64 White; 28 Black (20 Black Caribbean, 2 Black African, 6 Other Black); [8 Other]. | Not specified | Deliberate self harm | Black — | ?E, F, G, ?H, K |
| **Kramer 2000 [50]** | 1 | London, 1991-92 | Retrospective case notes analysis: 183 children: 102 White (74 British White; 28 White minority); 18 Black; 14 South Asian; [49 Mixed/Other] | Not specified | Referral to CAMHS | Black ↓*; South Asian —* | ?E, F, G, K |
| **Jayarajan 2001 [51]** | 3 | Birmingham, 1998 | Retrospective case notes analysis: 1068 children: 821 White; 116 Black (60 Black Caribbean, 4 Black African, 52 Black Other); 17 Indian; 71 Pakistani; 17 Bangladeshi; [44 Other] | Other method | Referral to CAMHS | Black ↑*; Indian ↓*; Pakistani ↓*; Bangladeshi ↓* | E, F, G, H, K |
| **Lamb 2002 [52]** | 1 | London, 1997 | Retrospective case notes analysis.: 444 children (380 from Tower Hamlets): 218 Bangladeshi (216 from Tower Hamlets); 316 non-Bangladeshi (254 from Tower Hamlets) | Not specified | 1) Referral to CAMHS 2) Proportional morbidity for referral reason | Tower Hamlets Bangladeshi ↓*; All Bangladeshi PM: behavioural —; emotional —; hyperactivity —; DSH —; psychosis — | ?E, G, K |
| **Willis 2002 [53]** | 3 | Manchester, 1999-2000 | Retrospective case notes analysis: 192 children aged 2-17: 141 White; 22 Black; 15 South Asian; [14 Mixed race/Other] | Not specified | Referral to CAMHS | Black —*; South Asian —* | ?E, F, G, ?H, K |
| **Bhugra, Thompson 2003 [54]** | 1 | London, 1994-95 | Retrospective case notes analysis: 61 children age 10-?19: 46 White British/Irish; 15 South Asian | Reported by child | Deliberate self harm | South Asian —* | F, G, ?H, K |
| **Messent 2003 [55]** | 1 | London, 1997 | Retrospective case notes analysis: 627 children aged 0-18: 352 White British; 36 Black Caribbean/Black British; 33 Black African; 206 Bangladeshi | Not specified | Referral to CAMHS | Black Caribbean —*;Black African —*; Bangladeshi ↓* | ?E, G, K |
| **Minnis 2003 [56]** | 1 | Glasgow, 2000-01† | Retrospective case notes analysis: 219 children: 17 South Asian; 212 non-South Asian | Not specified | Attendance at CAMHS | South Asian ↓** | ?E, F, G, ?H, K |
| **Tolmac 2004 [57]** | 1 | London, 2001 | Cross-sectional: 110 children aged 13-17: 66 White; 21 Black; 11 Asian; [12 Other] | Not specified | CAMHS in-patient with 1) non-psychotic or 2) psychotic disorder | Non-psychotic disorders: Black ↓*; South Asian ↓*. Psychotic disorders: Black ↑*; South Asian —* | ?E, F, G, K (non-psychotic disorders)/L(psychotic disorders) |
| **Hackett 2004 [58]** | 3 | Manchester, 2002-03 | Retrospective case notes analysis: 320 children aged 2-17: 205 White; 35 Black; 58 South Asian; [22 Mixed race/Other] | Not specified | Referral to CAMHS | Black ↑*; South Asian ↑* | ?E, F, G, ?H, K |
| **See notes to Table 4.** | | | | | | | |

# References

1. Bagley, C., Deviant behaviour in English and West Indian schoolchildren. Research in Education, 1972. 8: p. 47-55.

2. Rutter, M., et al., Children of West Indian immigrants. I. Rates of behavioural deviance and of psychiatric disorder. J Child Psychol Psychiatry, 1974. 15(4): p. 241-62.

3. Rutter, M., et al., Children of West Indian immigrants. III. Home circumstances and family patterns. J Child Psychol Psychiatry, 1975. 16(2): p. 105-23.

4. Kallarackal, A.M. and M. Herbert, The happiness of Indian immigrant children. New Society, 1976. 26 February: p. 422-424.

5. Cochrane, R., Psychological and behavioural disturbance in West Indians, Indians and Pakistanis in Britain: a comparison of rates among children and adults. Br J Psychiatry, 1979. 134: p. 201-10.

6. Earls, F. and N. Richman, The prevalence of behavior problems in three-year-old children of West Indian-Born parents. J Child Psychol Psychiatry 1980. 21(2): p. 99-106.

7. Osborn, A.F. and N.R. Butler, Ethnic minority children : a comparative study from birth to five years : a report of the Child Health and Education Study (1970 Birth Cohort). 1985, London: Commission for Racial Equality.

8. Hackett, L., R. Hackett, and D.C. Taylor, Psychological disturbance and its associations in the children of the Gujarati community. J Child Psychol Psychiatry, 1991. 32(5): p. 851-6.

9. Mumford, D.B., A.M. Whitehouse, and M. Platts, Sociocultural correlates of eating disorders among Asian schoolgirls in Bradford. Br J Psychiatry, 1991. 158: p. 222-8.

10. Newth, S.J. and J. Corbett, Behaviour and emotional problems in three-year-old children of Asian parentage. J Child Psychol Psychiatry, 1993. 34(3): p. 333-52.

11. Ahmad, S., G. Waller, and C. Verduyn, Eating attitudes among Asian schoolgirls: the role of perceived parental control. Int J Eat Disord, 1994. 15(1): p. 91-7.

12. McCourt, J. and G. Waller, Developmental role of perceived parental control in the eating psychopathology of Asian and Caucasian schoolgirls. Int J Eat Disord, 1995. 17(3): p. 277-82.

13. Waller, G., M. Coakley, and L. Richards, Bulimic attitudes among Asian and Caucasian schoolgirls. European Eating Disorders Review, 1995. 3(1): p. 24-34.

14. Marks, F.M., Symptoms in children of British and of Bangladeshi parents measured by the Rutter B2 Questionnaire. Ethn Health, 1997. 2(3): p. 255-9.

15. Nikapota, A., et al., Development of Culturally Appropriate Child Mental Health Services: Perceptions and Use of Services. , in Unpublished report to the Department of Health. 1998: London.

16. Nazroo, J., et al., Children's Health, in Health Survey for England: the Health of Minority Ethnic Groups, '99, Office for National Statistics, Editor. 1999, Stationery Office: London.

17. Meltzer, H., et al., Mental health of children and adolescents in Great Britain. 2000, London: The Stationery Office.

18. Ford, T., R. Goodman, and H. Meltzer, The British Child and Adolescent Mental Health Survey 1999: the prevalence of DSM-IV disorders. J Am Acad Child Adolesc Psychiatry, 2003. 42(10): p. 1203-11.

19. Ford, T., R. Goodman, and H. Meltzer, The relative importance of child, family, school and neighbourhood correlates of childhood psychiatric disorder. Soc Psychiatry Psychiatr Epidemiol, 2004. 39(6): p. 487-96.

20. Evans, R., Ethnic differences in ADHD and the mad/bad debate. Am J Psychiatry 2004. 161(5): p. 932; author reply 932.

21. Furnham, A. and S. Adam-Saib, Abnormal eating attitudes and behaviours and perceived parental control: a study of white British and British-Asian school girls. Soc Psychiatry Psychiatr Epidemiol, 2001. 36(9): p. 462-70.

22. Thomas, C.L., A.C. James, and M.O. Bachmann, Eating attitudes in English secondary school students: Influences of ethnicity, gender, mood, and social class. International Journal of Eating Disorders, 2002. 31(1): p. 92-96.

23. Bhugra, D. and K. Bhui, Eating disorders in teenagers in East London: A survey. European Eating Disorders Review, 2003. 11(1): p. 46-57.

24. Atzaba-Poria, N., A. Pike, and M. Barrett, Internalising and externalising problems in middle childhood: A study of Indian (ethnic minority) and English (ethnic majority) children living in Britain. International Journal of Behavioral Development, 2004. 28(5): p. 449-460.

25. Atzaba-Poria, N. and A. Pike, Why do ethnic minority (Indian) children living in Britain display more internalizing problems than their English peers? The role of social support and parental style as mediators. International Journal of Behavioral Development., 2005. 29(6): p. 532-540.

26. Atzaba-Poria, N. and A. Pike, Are ethnic minority adolescents at risk for problem behaviour? Acculturation and intergenerational acculturation discrepancies in early adolescence. British Journal of Developmental Psychology, Submitted.

27. Stansfeld, S.A., et al., Ethnicity, social deprivation and psychological distress in adolescents: school-based epidemiological study in east London. Br J Psychiatry, 2004. 185: p. 233-8.

28. Klineberg, E., Social support, ethnicity and mental health in adolescents. Soc Psychiatry Psychiatr Epidemiol., 2006. 41(9): p. 755-760.

29. Fagg, J., et al., Psychological distress among adolescents, and its relationship to individual, family and area characteristics in East London. Soc Sci Med, 2006. 63(3): p. 636-48.

30. Edmunds, S., et al., Child Health Assessment at School Entry (CHASE) project: evaluation in 10 London primary schools. Child Care Health Dev, 2005. 31(2): p. 143-54.

31. Flouri, E., Father's Involvement and Psychological Adjustment in Indian and White British Secondary School Age Children. Child and Adolescent Mental Health, 2005. 10(1): p. 32-39.

32. Frosh, S., et al., Prevalence of Emotional and Behavioural Disorders Among Strictly Orthodox Jewish Children in London. Clinical Child Psychology and Psychiatry, 2005. 10(3): p. 351-368.

33. Green, H., et al., Mental health of children and young people in Great Britain, 2004. 2005: Palgrave MacMillan.

34. Berry, J., et al., Immigrant Youth in Cultural Transition. Acculturation, Identity, and Adaptation Across National Contexts. 2006, Mahwah, NJ, US: Lawrence Erlbaum Associates.

35. Fuller, E., Children's health, in The health of minority ethnic groups, Volume 1, K. Sproston and J. Mindell, Editors. 2006, The Information Centre: Leeds.

36. Scott, S., T. O'Connor, and A. Futh, What makes parenting programmes work in disadvantaged areas? : the PALS trial. 2006, York Joseph Rowntree Foundation.

37. Wardle, J., et al., Depression in adolescent obesity: cultural moderators of the association between obesity and depressive symptoms. Int J Obes (Lond), 2006. 30(4): p. 634-43.

38. Laurens, K.R., et al., Psychotic-like experiences and other antecedents of schizophrenia in children aged 9-12 years: a comparison of ethnic and migrant groups in the United Kingdom. Psychol Med, 2007. 15: p. 1-9.

39. Maynard, M.J. and S. Harding, Ethnic differences in psychological well-being in adolescence in the context of time spent in family activities. Submitted.

40. Maynard, M.J., S. Harding, and H. Minnis, Psychological well-being in Black Caribbean, Black African, and White adolescents in the UK Medical Research Council DASH study. Soc Psychiatry Psychiatr Epidemiol, 2007. 42(9): p. 759-69.

41. Graham, P.J. and C.E. Meadows, Psychiatric disorder in the children of West Indian immigrants. J Child Psychol Psychiatry, 1967. 8(2): p. 105-16.

42. Taylor, E.A. and S.A. Stansfeld, Children who poison themselves. I. A clinical comparison with psychiatric controls. Br J Psychiatry, 1984. 145: p. 127-32.

43. Glover, G., F. Marks, and M. Nowers, Parasuicide in young Asian women. British Journal of Psychiatry., 1989. 154: p. 271-272.

44. Stern, G., D. Cottrell, and J. Holmes, Patterns of attendance of child psychiatry out-patients with special reference to Asian families. Br J Psychiatry, 1990. 156: p. 384-7.

45. Jawed, S.H., A survey of psychiatrically ill Asian children. Br J Psychiatry, 1991. 158: p. 268-70.

46. McGibben, L., et al., Deliberate self-poisoning in Asian and Caucasian 12-15-year-olds. Br J Psychiatry, 1992. 161: p. 110-2.

47. Goodman, R. and H. Richards, Child and adolescent psychiatric presentations of second-generation Afro-Caribbeans in Britain. Br J Psychiatry, 1995. 167(3): p. 362-9.

48. Roberts, N. and D. Cawthorpe, Immigrant child and adolescent psychiatric referrals: a five-year retrospective study of Asian and Caucasian families. Can J Psychiatry, 1995. 40(5): p. 252-6.

49. Goddard, N., F. Subotsky, and E. Fombonne, Ethnicity and adolescent deliberate self-harm. J Adolesc, 1996. 19(6): p. 513-21.

50. Kramer, T., N. Evans, and M.E. Garralda, Ethnic diversity among Child and Adolescent (CAP) clinic attenders. Child Psychology and Psychiatry, 2000. 5(4): p. 169-175.

51. Jayarajan, U., The Demographic Profile of the Children and Young People Referred to and Seen by Birmingham CAMHS. Unpublished report. 2001.

52. Lamb, G., A. Anfield, and A. Sheeran, Access to a child mental health service. A comparison of Bangladeshi and non-Bangladeshi families. Psychiatric Bulletin, 2002. 26: p. 15-18.

53. Willis, J., Audit of the Ethnicity of Referrals to a Child and Adolescent Mental Health, in South Asian Child and Adolescent Mental Health Service Development. Final Report. 2004, Unpublished report from the Manchester South Asian Child and Adolescent Mental Health Project: Manchester.

54. Bhugra, D., et al., Inception rates of deliberate self-harm among adolescents in West London. Int J Soc Psychiatry, 2003. 49(4): p. 247-50.

55. Messent, P. and M. Murrell, Research leading to action: a study of accessibility of a CAMH service to ethnic minority families. Child and Adolescent Mental Health, 2003. 8(3): p. 118-124.

56. Minnis, H., et al., Cultural and language mismatch: clinical complications. Clinical child psychology and psychiatry, 2003. 8(2): p. 179-186.

57. Tolmac, J. and M. Hodes, Ethnic variation among adolescent psychiatric in-patients with psychotic disorders. Br J Psychiatry, 2004. 184: p. 428-31.

58. Hackett, L. and J. Patel, Audit of Child and Adolescent Psychiatry referrals to the Winnicott centre, in South Asian Child and Adolescent Mental Health Service Development. Final Report. 2004, Unpublished report from the Manchester South Asian Child and Adolescent Mental Health Project: Manchester.
