# Supplementary material for: Child mental health differences amongst ethnic groups in Britain: a systematic review
Source: BMC Public Health. 2008 Jul 25;8:258. doi: 10.1186/1471-2458-8-258 (PMC2515844; doi:10.1186/1471-2458-8-258)
Supplement: Additional file 3 — Full description of excluded studies. References for excluded studies, with a more detailed description of why they were excluded: expands on the information in Figure 1. [file 1471-2458-8-258-S3.doc]

# Additional file 3 – Full details of excluded studies

## Table 3: Studies not included in review, with reasons for non-retrieval or non-inclusion

| **Reason for non-retrieval or non-inclusion (see Figure 1)** | **Author, date** | **Details** |
| --- | --- | --- |
| **Unable locate or research described never carried out** | Arnone [1] | Apparently unpublished research proposal, author(s) could not be contacted. |
|  | Minnis in press [2] | Unpublished study which had been cited [in 3] but where the authors were not clear what study was meant [4]. |
|  | Ramjee [5] | Apparently unpublished research proposal, author(s) could not be contacted. |
|  | Skinner [6] | Apparently unpublished research proposal, author(s) could not be contacted. |
|  | Walker 1968 [7] | Unpublished, could not be located |
|  | Zaineb [8] | Apparently unpublished research proposal, author(s) could not be contacted. |
|  | Zietlin [9] | Apparently unpublished research proposal, author(s) could not be contacted. |
| **Data collection/analysis not completed before deadline.** | Hodes [10] | Data collection or analysis not completed before the deadline |
|  | Kelly [11] | Data collection or analysis not completed before the deadline |
|  | Laurens [12] | Data collection or analysis not completed before the deadline |
|  | Muthulagu [13] | Data collection or analysis not completed before the deadline |
|  | Truman [14] | Data collection or analysis not completed before the deadline |
| **Sampled from specially selected groups** | Chowdhury 2005 [15] | Sampled from a medium secure health service unit, mostly serving adolescents convicted of a criminal offence. |
|  | Kelsall 1995 [16] | Sampled from a medium secure health service unit, mostly serving adolescents convicted of a criminal offence. |
|  | Leavy 2004 [17] | Compares refugee to non-refugee children |
|  | Morita 1987 [18] | Children of expatriate Japanese businessmen, temporarily studying in Japanese schools in Britain. |
|  | Ullah 1985 [19] | Unemployed 17-year olds receiving benefits. |
| **Did not contain an eligible ethnic group** | Ahmad 1994b[20] | South Asian population subdivided by religious group (Hindu and Muslim). Note: results on female sample are presented in Ahmad 1994a |
|  | Boeing 2007 [21] | Only ethnic comparison is European vs. non-European origin. |
|  | Burt 2004 [22] | Only ethnic comparison is White British vs. non-White British. |
|  | Flouri 2006 [23] | Only ethnic comparison is White vs. non-White. |
|  | Gowers 1993 [24] | Only ethnic comparison is White British vs. non-White British. |
|  | Mears 2003 [25] | Only ethnic comparison is White vs. non-White. |
|  | O'Herlihy 2001 [26] | Only ethnic comparison is White vs. non-White. |
|  | Schmidt 1992 [27] | Only 'ethnic' comparison is according to membership of an ethnically heterogenous group loosely defined by 'cultural stress' |
|  | Sonuga-Barke 2000 [28] | Compares Hindu children with Muslim children, this division cutting across families India, Pakistan or East Africa as a place of origin. |
|  | Stevenson 1985 [29] | Only ethnic comparison is immigrant vs. non-immigrant. |
| **Contained no eligible ethnic comparison** | Lindsey 2003 [30] | Inadequate information about the small external general population comparison group to determine when and where it was collected. |
|  | Nicol 1971 [1st study] [31] | Comparison within Black-Caribbeans, according to whether they were born inside the UK or not. |
|  | Shah 1995 [32] | Comparison within Pakistanis, according to whether they were from extended or nuclear families. |
| **Did not give the number of participants per ethnic group** | Dex 2007 [33] | Number of children of each ethnic group not given |
|  | Cochrane 1979 [2nd Study] [34] | Number of children of each ethnic group not given |
|  | Furnham 1994 [35] | Inconsistencies between number of children as reported by ethnic group and as reported by religion, which the authors could not clarify [36]. |
|  | Hillier 1994 [37] | Brief reference to an underrepresentation of Bangladeshis at their child and adolescent mental health clinic in passing. |
|  | Vyas 1998 [38] | Brief poster abstract in which number of children of each ethnic group not given. |
| **Did not measure a mental health outcome of interest** | Moran 2007 [39] | Mental health outcome outside the scope of this review (psychopathy). |
|  | Harris 2001 [40] | Evaluation of service delivery |
|  | Hill 1995 [41] | Measures eating style/behaviour, but not eating disorders. |
|  | Ogden 1998 [42] | Measures eating style/behaviour, but not eating disorders. |
| **Did not use a valid/validated mental health measure** | Bhatnagar 1970 [43] | Non-validated 'adjustment scale' constructed for the study. |
|  | Dosanjh 1976 [44] | Non-validated mental health and temperament scales constructed for the study. |
|  | Dosanjh 1996 [45] | Non-validated mental health and temperament scales constructed for the study. |
|  | Hackett 1993 [46] | Non-validated question on 'temper tantrums', constructed for the study. |
|  | Schools Council 1970 [47] | Measures social adjustment using the Bristol Social Adjustment Guide, [48] judged not to be satisfactorily validated as a mental health measure. |
|  | Hawton 2002 [49] | Did not use a validated instrument for assessing deliberate self harm |
|  | Marchant 2006 [50] | Mental health status assessed using school registries of special educational needs for autistic spectrum disorder - i.e. an administrative registry not created for the purposes of mental health research. |
| **Not of an eligible study type or size** | Bhugra 2002 [51] | Compares reasons and risk factors among those who have committed deliberate self-harm. |
|  | Earls 1980b [52] | Compares mental health prognosis. |
|  | Handy 1991 [53] | Compares methods of self-poisoning and risk factors among those admitted with deliberate self-harm. |
|  | Holden 1988 [54] | Compares symptoms within matched samples of children with anorexia. |
|  | Kingsbury 1994 [55] | Compares symptoms and risk factors within children who have committed deliberate self-harm. |
|  | Nicol 1971 [2nd study] [31] | Compares mental health prognosis. |
|  | Nicol 1971 [3rd study] [31] | Compares symptoms within children with behavioural disorder |
|  | Sonuga-Barke 1993 [1st study] [56] | Compares teacher's assessment of hyperactivity against objective measures. |
|  | Sonuga-Barke 1993 [2nd study] [56] | Compares teacher's assessment of hyperactivity against objective measures. |
|  | Tareen 2005 [57] | Comparing risk factors and symptoms within girls presenting with eating disorder. |
|  | Vostanis 2003 [58] | Compares service use among children with conduct disorder |
| **Referral rates not calculated in relation to a specified base population** | Bendall 1972 [59] | No explicit comparison of number of diagnoses of anorexia in South Asians with a base population. |
|  | Fatimilehin 1998 [60] | No explicit comparison of number of referrals for Black and non-Black children with a base population. |
|  | Subotsky 1990 [61] | No explicit comparison of number of referrals from each ethnic group with a base population. |
| **Could not extract an analysis by ethnic group for an outcome of interest** | Best 2006 [62] | Does not present mental health outcome by ethnic group. |
|  | Daryanani 2001 [63] | Single simultaneous comparison of the proportional morbidity for 19 mental health outcomes, only 7 of which are within the scope of this review and which cannot be extracted from those which are not. |
|  | Shams 1995 [64] | Mental health outcome of interest (GHQ) not compared between ethnic groups, but used only as a covariate. |
| **Duplicate publications containing no new information.** | Atzaba-Poira 2004b [65] | Duplicates Atzaba Poira 2004a [66] |
|  | Bhui 2005 [67] | Duplicates Stansfield 2004 [68] |
|  | Bhugra 2004 [69] | Duplicates Bhugra 2003b [70] |
|  | Deater-Deckard 2004 [71] | Duplicates Atzaba Poira 2004a [66] |
|  | Earls 1982 [72] | Duplicates Earls 1980a [73] |
|  | Hackett 1994 [74] | Duplicates Hackett 1991 [75] |
|  | Mumford 1988 [76] | Duplicates Mumford 1991 [77] |

# References

1. Arnone D: **Ethnic influence in the diagnosis of autism**. [Study description in NRR database].

2. Minnis H, Kelly E, Bradby H: *The use of child psychiatry by South Asian Families in South Glawgow (cited in Malek, M., Meeting the needs of minority ethnic groups in the UK, in Mental Health Services for Minority Ethnic Children and Adolescents, M. Malek and C. Joughin, Editors. 2004, Jessica Kingsley: London).* In press.

3. Malek M: **Meeting the needs of minority ethnic groups in the UK**. In *Mental Health Services for Minority Ethnic Children and Adolescents*. Edited by Malek M, Joughin C. London: Jessica Kingsley; 2004.

4. Minnis H: **re: request for information [2]**. Edited by Goodman A; 22nd March 2007.

5. Ramjee MJ: **An investigation into the increase from 1983 to 2003 (20 years) of reported cases of psychosis in young girls of South Asian ethnicity in Tier 4 of the CAMHS on Ward 3 at Birmingham Children's Hospital** [Study description in NRR database].

6. Skinner A: **An Epidemiological study of deliberate self-harm in an inner-city, multi-ethnic adolescent population**. [Study description in NRR database].

7. Walker M: **West Indian Children in school. Unpublished report**. [Cited in Nicol, A.R., Psychiatric disorder in the children of Caribbean immigrants. J Child Psychol Psychiatry, 1971. 12(4): p. 273-87]; 1968.

8. Zaineb DA: **Differences in factors associated with deliberate self-harm in children and adolescents: A transcultural comparison**. [Study description in NRR database].

9. Zietlin H: **Risk and Protective Factors in adolescents who attempt suicide: Differences across ethnic groups**. [Study description in NRR database].

10. Hodes M: **West London adolescent psychosis study**. [Study description in NRR database].

11. Kelly Y: **re: MCS Mental Health**. Edited by Goodman A; 22nd May 2007.

12. Laurens KR: **RE: Antecedents of schizophrenia in children from differentethnicgroups**. Edited by Goodman A; 19th May 2007.

13. Muthalagu S: **Eatinng Attitudes and Behaviours among Adolescents in East London - Relationship with Etnicity**. [Study description in NRR database].

14. Truman J: **RE: Child mental health and ethnicity - request for help locating studies**. Edited by Goodman A; 22nd May 2007.

15. Chowdhury NA, Whittle N, McCarthy K, Bailey S, Harrington R: **Ethnicity and its relevance in a seven-year admission cohort to an English national adolescent medium secure health service unit**. *Crim Behav Ment Health* 2005, **15**(4):261-272.

16. Kelsall M, Dolan M, Bailey S: **Violent incidents in an adolescent forensic unit**. *Med Sci Law* 1995, **35**(2):150-158.

17. Leavey G, Hollins K, King M, Barnes J, Papadopoulos C, Grayson K: **Psychological disorder amongst refugee and migrant schoolchildren in London**. *Soc Psychiatry Psychiatr Epidemiol* 2004, **39**(3):191-195.

18. Morita H: **Behavioural deviance in Japanese children attending school in England**. *Acta Paediatr Jpn* 1987, **29**(2):233-240.

19. Ullah P, Banks M, Warr P: **Social support, social pressures and psychological distress during unemployment**. *Psychological Medicine* 1985, **15**(2):283-295.

20. Ahmad S, Waller G, Verduyn C: **Eating Attitudes and Body Satisfaction among Asian and Caucasian Adolescents**. *Journal of Adolescence* 1994, **17**(5):461-470.

21. Boeing L, Murray V, Pelosi A, McCabe R, Blackwood D, Wrate R: **Adolescent-onset psychosis: prevalence, needs and service provision**. *Br J Psychiatry* 2007, **190**:18-26.

22. Burt KB, Hay DF, Pawlby S, Harold G, Sharp D: **The prediction of disruptive behaviour disorders in an urban community sample: the contribution of person-centred analyses**. *J Child Psychol Psychiatry* 2004, **45**(6):1159-1170.

23. Flouri E: **Non-resident fathers' relationships with their secondary school age children: determinants and children's mental health outcomes**. *J Adolesc* 2006, **29**(4):525-538.

24. Gowers S, Entwistle K, Cooke N, Okpalugo B, Kenyon A: **Social and family factors in adolescent psychiatry**. *J Adolesc* 1993, **16**(4):353-366.

25. Mears A, White R, O'Herlihy A, Worrall A, Banerjee S, Jaffa T, Hill P, Lelliott P: **Characteristics of the Detained and Informal Child and Adolescent Psychiatric In-Patient Populations**. *Child and Adolescent Mental Health* 2003, **8**(3):131-134.

26. O’Herlihy A, Worral A, Banerjee S, Jaffa T, Hill P, Mears A, Brook H, Scott A, White R, Nikoloaou V *et al*: *National In-patient Child and Adolescent Psychiatry Study (NICAPS): Final Report to the Department of Health*. London: Royal College of Psychiatrists’ Research Unit; 2001.

27. Schmidt U, Hodes M, Treasure J: **Early onset bulimia nervosa: who is at risk? A retrospective case-control study**. *Psychol Med* 1992, **22**(3):623-628.

28. Sonuga-Barke EJ, Mistry M: **The effect of extended family living on the mental health of three generations within two Asian communities**. *Br J Clin Psychol* 2000, **39 ( Pt 2)**:129-141.

29. Stevenson J, Richman N, Graham P: **Behaviour problems and language abilities at three years and behavioural deviance at eight years**. *J Child Psychol Psychiatry* 1985, **26**(2):215-230.

30. Lindsey C, Frosh S, Loewenthal K, Spitzer E: **Prevalence of Emotional and Behavioural Disorders Among Strictly Orthodox Jewish Pre-School Children in London**. *Clinical Child Psychology and Psychiatry* 2003, **8**(4):459-472.

31. Nicol AR: **Psychiatric disorder in the children of Caribbean immigrants**. *J Child Psychol Psychiatry* 1971, **12**(4):273-287.

32. Shah Q, Sonuga-Barke E: **Family structure and the mental health of Pakistani Muslim mothers and their children living in Britain**. *Br J Clin Psychol* 1995, **34 ( Pt 1)**:79-81.

33. Dex S, Ward K: **Parental care and employment in early childhood: analysis of the Millennium Cohort Study (MCS) sweeps 1 and 2, Research Report**. Manchester: Equal Opportunities Commission; 2007.

34. Cochrane R: **Psychological and behavioural disturbance in West Indians, Indians and Pakistanis in Britain: a comparison of rates among children and adults**. *Br J Psychiatry* 1979, **134**:201-210.

35. Furnham A, Patel R: **The eating attitudes and behaviours of Asian and British schoolgirls: a pilot study**. *Int J Soc Psychiatry* 1994, **40**(3):214-226.

36. Furnham A: **RE: request for information**. Edited by Goodman A; 16th February 2007.

37. Hillier S, Loshak R, Ralman S, Marks F: **An evaluation of child psychiatric services for Bangladeshi parents**. *Journal of Mental Health* 1994, **3**:327-337.

38. Vyas I, Stretch D, Dawson S, Nicol AR: **Two cultures in one city. Similarities and differences in child rearing between native and Indian families of three-year-olds.** In Poster presented at the 14th International Congress of the International Association for Child and Adolescent Psychiatry and Allied Professions 1998.

39. Moran P, Ford T, Butler G, Goodman R: **Callous and unemotional traits in children and adolescents living in Great Britain**. In press.

40. Harris R, Hardman E: **Developing and Evaluating Community Mental Health Services: The Bangladeshi Community, Assessment of Need.** *Journal of Clinical Excellence* 2001, **3**(2):69-73.

41. Hill AJ, Bhatti R: **Body shape perception and dieting in preadolescent British Asian girls: links with eating disorders**. *Int J Eat Disord* 1995, **17**(2):175-183.

42. Ogden J, Elder C: **The role of family status and ethnic group on body image and eating behavior**. *International Journal of Eating Disorders* 1998, **23**(3):309-315.

43. Bhatnagar J: *Immigrants at school*. London: Cornmarket press; 1970.

44. Dosanjh JS: **A comparative study of Punjabi and English child rearing practices with special reference to lower juniors (7-9 years)** University of Nottingham, Psychology; 1976.

45. Dosanjh JS, Ghuman P: *Child-rearing in ethnic minorities*. Avon: Multilingual Matters Ltd; 1996.

46. Hackett L, Hackett R: **Parental ideas of normal and deviant child behaviour. A comparison of two ethnic groups**. *Br J Psychiatry* 1993, **162**:353-357.

47. Schools Council: *Teaching English to West Indian children : the research stage of the project*. London: Evans/Methuen Educational; 1970.

48. Stott DH: *The social-adjustment of children: Manual to the Bristol Social-Adjustment Guides*. London: University of London press; 1963.

49. Hawton K, Rodham K, Evans E, Weatherall R: **Deliberate self harm in adolescents: self report survey in schools in England**. *Bmj* 2002, **325**(7374):1207-1211.

50. Marchant P, Hussain A, Hall K: **Autistic Spectrum Disorders and Asian children**. *British Journal of Educational Studies* 2006, **54**(2):230-244.

51. Bhugra D, Singh J, Fellow-Smith E, Bayliss C: **Deliberate self-harm in adolescents. A case note study among two ethnic groups**. *European Journal of Psychiatry* 2002, **16**(3):145-151.

52. Earls F, Richman N: **Behavior problems in pre-school children of West Indian-born parents: a re-examination of family and social factors**. *J Child Psychol Psychiatry* 1980, **21**(2):107-117.

53. Handy S, Chithiramohan RN, Ballard CG, Silveira WR: **Ethnic differences in adolescent self-poisoning: a comparison of Asian and Caucasian groups**. *J Adolesc* 1991, **14**(2):157-162.

54. Holden NL, Robinson PH: **Anorexia nervosa and bulimia nervosa in British blacks**. *Br J Psychiatry* 1988, **152**:544-549.

55. Kingsbury S: **The Psychological and Social Characteristics of Asian Adolescent Overdose**. *Journal of Adolescence* 1994, **17**(2):131-135.

56. Sonuga-Barke EJS, Minocha K, Taylor EA, Sandberg S: **Inter-ethnic bias in teachers' ratings of childhood hyperactivity**. *British Journal of Developmental Psychology* 1993, **11**(2):187-200.

57. Tareen A, Hodes M, Rangel L: **Non-fat-phobic anorexia nervosa in British South Asian adolescents**. *Int J Eat Disord* 2005, **37**(2):161-165.

58. Vostanis P, Meltzer H, Goodman R, Ford T: **Service utilisation by children with conduct disorders--findings from the GB National Study**. *Eur Child Adolesc Psychiatry* 2003, **12**(5):231-238.

59. Bendall P, Hamilton M, Holden N: **Eating disorders in Asian girls**. *British Journal of Psychiatry* 1991, **159**:441.

60. Fatimilehin IA, Coleman PG: **Appropriate services for African-Caribbean families: views from one community.** *Clinical Psychology Forum* 1998, **111**:6-11.

61. Subotsky F, Berelowitz G: **Consumer views at a community child guidance clinic**. *Newsletter of the Association for Child Psychology and Psychiatry* 1990, **12**(3):8-12.

62. Best D, Manning V, Gossop M, Gross S, Strang J: **Excessive drinking and other problem behaviours among 14-16 year old schoolchildren**. *Addict Behav* 2006, **31**(8):1424-1435.

63. Daryanani R, Hindley P, Evans C, Fahy P, Turk J: **Ethnicity and use of a child and adolescent mental health service**. *Child Psychology and Psychiatry Review* 2001, **6**(3):127-132.

64. Shams M, Williams R: **Differences in perceived parental care and protection and related psychological distress between British Asian and non-Asian adolescents**. *Journal of Adolescence* 1995, **18**:329-348.

65. Atzaba-Poria N, Pike A, Deater-Deckard K: **Do risk factors for problem behaviour act in a cumulative manner? An examination of ethnic minority and majority children through an ecological perspective**. *J Child Psychol Psychiatry* 2004, **45**(4):707-718.

66. Atzaba-Poria N, Pike A, Barrett M: **Internalising and externalising problems in middle childhood: A study of Indian (ethnic minority) and English (ethnic majority) children living in Britain**. *International Journal of Behavioral Development* 2004, **28**(5):449-460.

67. Bhui K, Stansfeld S, Head J, Haines M, Hillier S, Taylor S, Viner R, Booy R: **Cultural identity, acculturation, and mental health among adolescents in east London's multiethnic community**. *J Epidemiol Community Health* 2005, **59**(4):296-302.

68. Stansfeld SA, Haines MM, Head JA, Bhui K, Viner R, Taylor SJ, Hillier S, Klineberg E, Booy R: **Ethnicity, social deprivation and psychological distress in adolescents: school-based epidemiological study in east London**. *Br J Psychiatry* 2004, **185**:233-238.

69. Bhugra D, Thompson N, Singh J, Fellow-Smith E: **Deliberate self-harm in adolescents in West London: Socio-cultural factors**. *European Journal of Psychiatry* 2004, **18**(2):91-98.

70. Bhugra D, Thompson N, Singh J, Fellow-Smith E: **Inception rates of deliberate self-harm among adolescents in West London**. *Int J Soc Psychiatry* 2003, **49**(4):247-250.

71. Deater-Deckard K, Atzaba-Poria N, Pike A: **Mother- and father-child mutuality in Anglo and Indian British families: a link with lower externalizing problems**. *J Abnorm Child Psychol* 2004, **32**(6):609-620.

72. Earls F: **Cultural and national differences in the epidemiology of behavior problems of preschool children**. *Cult Med Psychiatry* 1982, **6**(1):45-56.

73. Earls F, Richman N: **The prevalence of behavior problems in three-year-old children of West Indian-Born parents**. *J Child Psychol Psychiatry* 1980, **21**(2):99-106.

74. Hackett L, Hackett R: **Child-rearing practices and psychiatric disorder in Gujarati and British children**. *British Journal of Social Work* 1994, **24**(2):191-202.

75. Hackett L, Hackett R, Taylor DC: **Psychological disturbance and its associations in the children of the Gujarati community**. *J Child Psychol Psychiatry* 1991, **32**(5):851-856.

76. Mumford DB, Whitehouse AM: **Increased prevalence of bulimia nervosa among Asian schoolgirls**. *Bmj* 1988, **297**(6650):718.

77. Mumford DB, Whitehouse AM, Platts M: **Sociocultural correlates of eating disorders among Asian schoolgirls in Bradford**. *Br J Psychiatry* 1991, **158**:222-228.
